# Development of MHz X-ray phase contrast imaging at the European XFEL

**DOI:** 10.1107/S160057752400986X

**Published:** 2025-01-01

**Authors:** Jayanath C. P. Koliyadu, Daniel Moško, Eleni Myrto Asimakopoulou, Valerio Bellucci, Šarlota Birnšteinová, Richard Bean, Romain Letrun, Chan Kim, Henry Kirkwood, Gabriele Giovanetti, Nerea Jardon, Janusz Szuba, Trey Guest, Andreas Koch, Jan Grünert, Peter Szeles, Pablo Villanueva-Perez, Fabian Reuter, Claus-Dieter Ohl, Mike Andreas Noack, Francisco Garcia-Moreno, Zuzana Kuglerová-Valdová, Libor Juha, Martin Nikl, Wataru Yashiro, Hitoshi Soyama, Daniel Eakins, Alexander M. Korsunsky, Jozef Uličný, Alke Meents, Henry N. Chapman, Adrian P. Mancuso, Tokushi Sato, Patrik Vagovič

**Affiliations:** a European XFEL GmbH Schenefeld Germany; b University of P. J. Šafárik, Kosice, Slovakia; c Synchrotron Radiation Research and NanoLund Lund University Sweden; d Department of Chemistry and Physics, La Trobe Institute for Molecular Science La Trobe University Melbourne Victoria3086 Australia; e Faculty of Natural Sciences, Institute for Physics Otto von Guericke University Magdeburg Universitätsplatz 2 39106Magdeburg Germany; f Institute of Materials Science and Technology, Technische Universität Berlin, Hardenbergstrasse 36, 10623Berlin, Germany; g Institute of Applied Materials Helmholtz-Zentrum Berlin für Materialien und Energie Hahn-Meitner-Platz 1 14109Berlin Germany; h FZU – Institute of Physics Czech Academy of Sciences Prague Czech Republic; i International Center for Synchrotron Radiation Innovation Smart (SRIS) Tohoku University Katahira 2-1-1, Aoba-ku Sendai Miyagi980-8577 Japan; j Institute of Multidisciplinary Research for Advanced Materials (IMRAM) Tohoku University Katahira 2-1-1, Aoba-ku Sendai Miyagi980-8577 Japan; k Department of Applied Physics, School of Engineering The University of Tokyo 7-3-1 Hongo, Bunkyo-ku Tokyo113-8656 Japan; l Department of Finemechanics Tohoku University 6-6-01 Aramaki, Aoba-ku Sendai980-8579 Japan; m Department of Engineering Science University of Oxford Parks Road OxfordOX1 3PJ United Kingdom; n Center for Free-Electron Laser Science (CFEL) Deutsches Elektronen-Synchrotron (DESY) Notkestrasse 85 22607Hamburg Germany; o The Hamburg Centre for Ultrafast Imaging, Luruper Chaussee 149, 22761Hamburg, Germany; p Department of Physics, Universität Hamburg, Luruper Chaussee 149, 22761Hamburg, Germany; r Diamond Light Source Ltd Harwell Science and Innovation Campus DidcotOX11 0DE United Kingdom; RIKEN SPring-8 Center, Japan

**Keywords:** megahertz sampling, X-ray phase contrast imaging, pulse-resolved imaging, indirect MHz X-ray detector, European XFEL

## Abstract

We report on recent developments that enable megahertz hard X-ray phase contrast imaging (MHz XPCI) experiments at the Single Particles, Clusters, and Biomolecules and Serial Femtosecond Crystallography (SPB/SFX) instrument of the European XFEL facility (EuXFEL). We describe the technical implementation of the key components, including an MHz fast camera and a modular indirect X-ray microscope system based on fast scintillators coupled through a high-resolution optical microscope, which enable full-field X-ray microscopy with phase contrast of fast and irreversible phenomena. The image quality for MHz XPCI data showed significant improvement compared with a pilot demonstration of the technique using parallel beam illumination, which also allows access to up to 24 keV photon energies at the SPB/SFX instrument of the EuXFEL. With these developments, MHz XPCI was implemented as a new method offered for a broad user community (academic and industrial) and is accessible via standard user proposals. Furthermore, intra-train pulse diagnostics with a high few-micrometre spatial resolution and recording up to 128 images of consecutive pulses in a train at up to 1.1 MHz repetition rate is available upstream of the instrument. Together with the diagnostic camera upstream of the instrument and the MHz XPCI setup at the SPB/SFX instrument, simultaneous two-plane measurements for future beam studies and feedback for machine parameter tuning are now possible.

## Abstract 2

The development of instrumentation as well as applications for megahertz X-ray phase contrast imaging at the Single Particles, Clusters, and Biomolecules and Serial Femtosecond Crystallography instrument of the European XFEL are introduced.

## 1. Introduction

Hard X-ray full-field imaging is an increasingly important tool for revealing structural information of samples related to broad scientific interests from materials science through fluidics, biological research and more (Cao *et al.*, 2020 ▸; San Lee *et al.*, 2013 ▸; Kim *et al.*, 2008 ▸; Sena *et al.*, 2022 ▸). Significant development of full-field X-ray microscopy techniques has been enabled by the availability of third- and fourth-generation synchrotron sources (Haensel, 1994 ▸; Galayda, 1995 ▸; Ueki & Yamamoto, 1999 ▸) owing to their performance, especially their high photon flux with a large spatial coherence. Over the last quarter of a century, thanks to those sources, the methods of X-ray microscopy, especially phase-contrast or phase-sensitive methods (Snigirev *et al.*, 1995 ▸; Cloetens *et al.*, 1996 ▸; David *et al.*, 2002 ▸; Momose *et al.*, 2003 ▸; Dierolf *et al.*, 2010 ▸), have been developed, now reaching spatial resolutions in the single-digit nanometre range (Bajt *et al.*, 2018 ▸; Matsuyama *et al.*, 2018 ▸). Although these developments were widely focused on improving spatial resolution, the temporal resolution is less developed. With the development of fast X-ray detectors, the pulsed nature of synchrotron sources has been explored and full-field imaging, synchronized to individual X-ray pulses, has been demonstrated. Pulse-synchronized full-field X-ray phase contrast imaging (XPCI) was pioneered at the Advanced Photon Source (Fezzaa & Wang, 2008 ▸) and was recently established at the European Synchrotron Radiation Facility (ESRF) (Olbinado *et al.*, 2017 ▸) at megahertz (MHz) sampling rates. Next-generation X-ray sources, namely the X-ray free-electron lasers (XFELs), provide X-ray pulses with even shorter duration, down to the femtosecond scale and, most importantly, with full or near-full coherent illumination with three orders of magnitude more photons per pulse. The full coherent illumination maximizes the phase contrast and this provide access to quantitative information after performing phase retrieval (Snigirev *et al.*, 1995 ▸; Paganin *et al.*, 2002 ▸). With these characteristics, XFELs have the potential to advance the capabilities of fast X-ray full-field imaging methods even further. Among all XFEL sources, only the European XFEL (EuXFEL) (Tschentscher *et al.*, 2017 ▸; Decking *et al.*, 2020 ▸) provides pulses at MHz repetition rates (up to 4.5 MHz), which enabled the first feasibility study and demonstration of MHz XPCI synchronized to individual X-ray pulses (Vagovič *et al.*, 2019 ▸). This feasibility study confirmed that EuXFEL can indeed be used as a unique X-ray source for the characterization of stochastic phenomena occurring in various systems with high spatial and temporal resolution. In this work, we describe the recent developments of the instrumentation for MHz XPCI and beam diagnostics developed and installed at the Single Particles, Clusters, and Biomolecules and Serial Femtosecond Crystallography (SPB/SFX) instrument (Mancuso *et al.*, 2019 ▸) of the EuXFEL and provide examples of applications. With these developments, a new method for the study of fast irreversible processes has been implemented and is available for a broad range of academic and industrial users, accessible via the EuXFEL standard proposal application process.

## 2. Instrumentation for MHz XPCI at the EuXFEL

**Figure 1:**
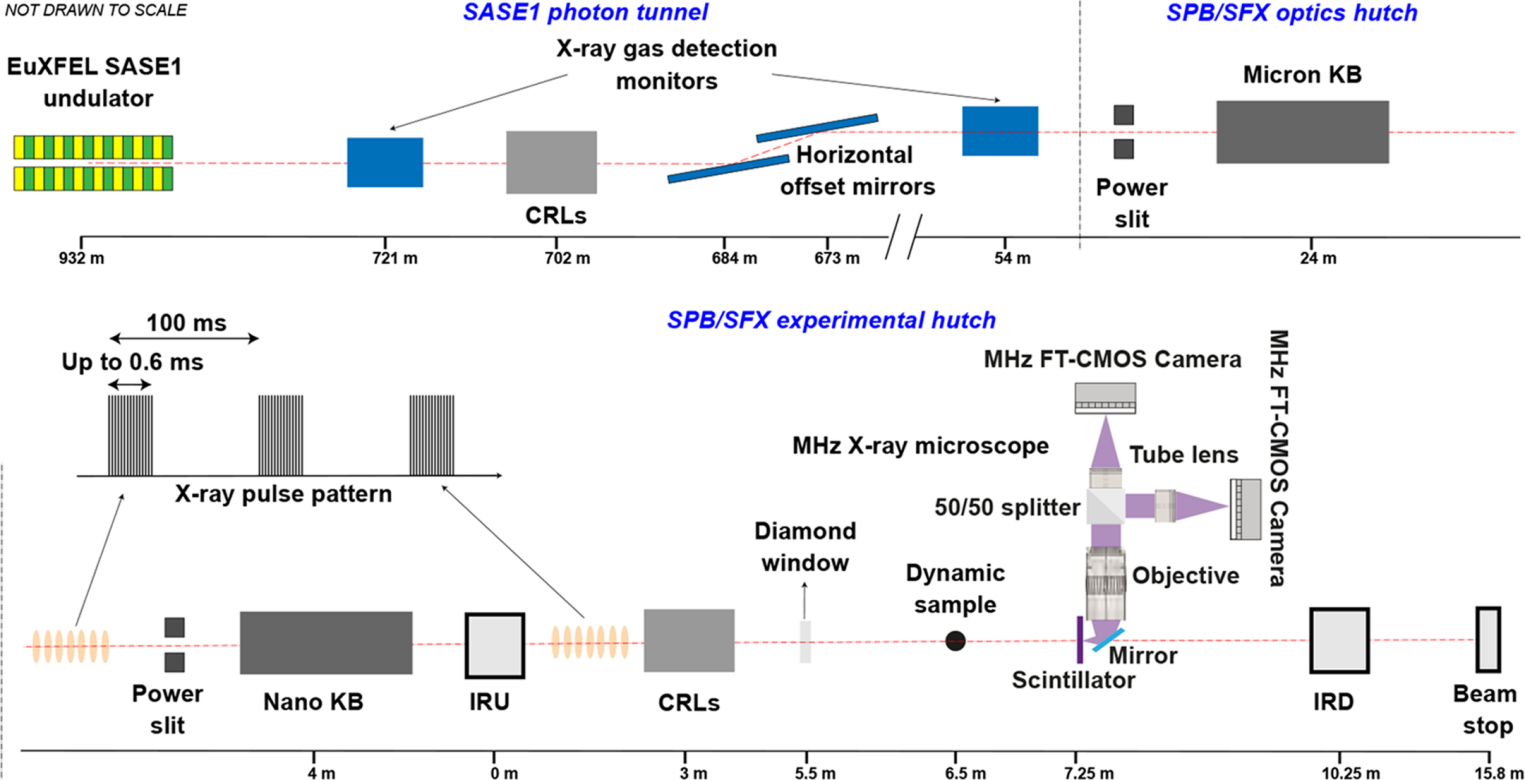
Schematic of the parallel beam configuration for MHz XPCI experiments at SPB/SFX. Following the horizontal offset mirrors, the beam passes through an X-ray gas detection monitor, which measure the pulse energy. It then passes through a set of power slits and, if out-coupled to air, a diamond window. The indirect detection microscopes are placed after a dynamic sample downstream of the diamond window. The focusing optics, KB mirrors and CRLs are shown in their respective positions relative to the MHz XPCI setup and are moved out of the X-ray beam for the parallel beam configuration. The CRLs in the photon tunnel are used to collimate the parallel beam to achieve higher flux density in cases where the number of photons per pulse is not enough to produce a good signal-to-noise ratio, especially for hard X-rays > 20 keV. The X-ray pulse structure is also shown.

MHz XPCI at EuXFEL has been pioneered and implemented at the scientific instrument SPB/SFX (Mancuso *et al.*, 2019 ▸; Vagovič *et al.*, 2019 ▸). The SPB/SFX instrument was primarily designed to study structures of biological molecules and single particles using serial femtosecond X-ray crystallography (Wiedorn *et al.*, 2018 ▸; Grünbein *et al.*, 2018 ▸; Yefanov *et al.*, 2019 ▸) or single-particle imaging (Sobolev *et al.*, 2020 ▸; Ayyer *et al.*, 2021 ▸). It is located at the SASE1 branch of the EuXFEL and a schematic of the instrument configuration with optical elements involved is shown in Fig. 1 ▸.

At SPB/SFX, there are two interaction regions – interaction region upstream (IRU) and interaction region downstream (IRD) – along the instrument. The MHz XPCI setup can be placed between IRU and IRD or downstream of IRD. The instrument can be optimized for different X-ray beam configurations, from different X-ray beam focusing configurations based on Kirkpatrick–Baez (KB) mirrors and compound refractive lenses (CRLs) (Bean *et al.*, 2016 ▸; Mancuso *et al.*, 2019 ▸) to a parallel beam configuration, by moving out the focusing optics from the beam, where only two horizontal offset mirrors are used to steer the beam through the instrument. Although we call the X-ray beam parallel, the beam has a source divergence on the order of a few microradians, resulting in a beam size of about 3 mm at the experimental hutch due to the almost 1 km distance from the source. This beam could be collimated using the CRLs in the tunnel to achieve a smaller beam size and higher flux density at the sample position. One set of KB mirrors can focus the beam down to approximately 2 µm and is therefore referred to as the micron KB. The other, called the nano KB, can focus down to 100 nm. These mirrors are used to focus the X-ray beam to IRU. The focused beam for IRD is delivered using refocusing of the micron KB beam using CRLs, allowing for two parallel diffraction-type experiments at IRU and IRD or a collimated beam using CRLs in the tunnel and focusing such beam using CRLs at the experimental hutch.

**Figure 2:**
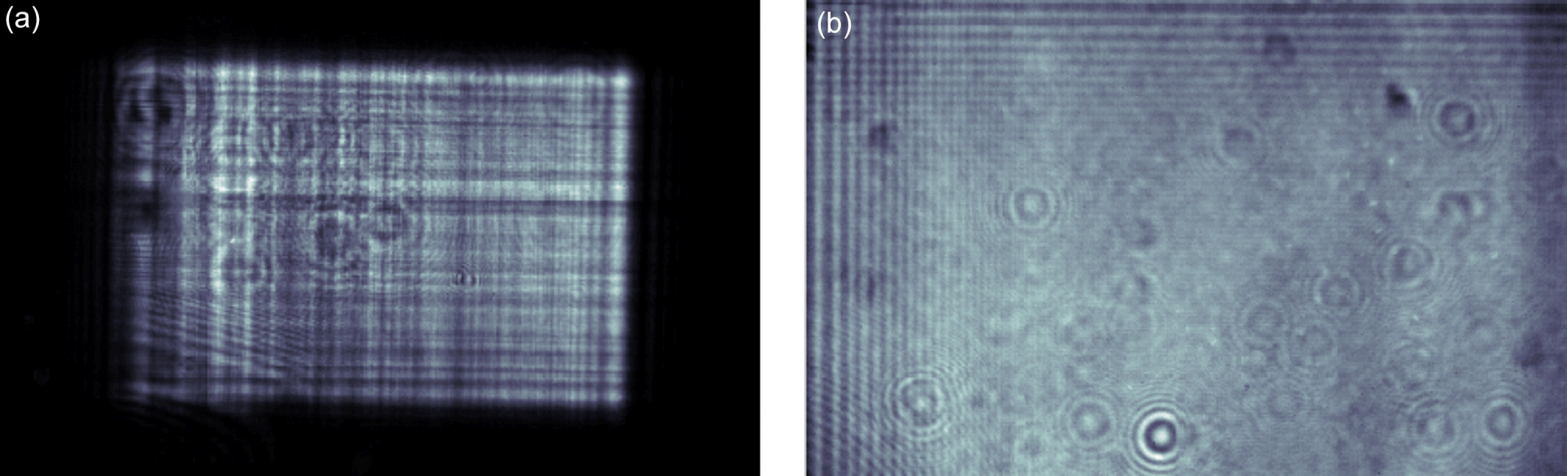
X-ray beam imaged using the MHz X-ray microscope with (*a*) the micron KB beam downstream of the focus and parallel beam illumination (*b*). The field of view in both cases is 1.28 × 0.8 mm. The Fresnel fringes on the top left in (*b*) arise from the power slit upstream of the IRU chamber and the round features in both images can be attributed to imperfections in the exit window. The measurement was carried out at a photon energy of 9.3 keV for both beam trajectories.

The first MHz XPCI experiments used the spent micron KB beam with the imaging setup placed several meters downstream of the focus position. However, the focusing mirrors introduce wavefront distortions that result in the appearance of vertical and horizontal streaks that act as high-frequency noise-obscuring image quality (Rack *et al.*, 2010 ▸). Therefore, by removing this optics from the beam path and using only horizontal offset mirrors the quality of illumination significantly improved, and such configuration also provided access to a larger beam size and higher photon energies above 16 keV, which are the design limits of the KB optics at the SPB/SFX instrument. A comparison of the KB beam and parallel beam is shown in Fig. 2 ▸.

The maximum achievable beam size of the parallel beam can reach up to ∼3 mm; however, in practice it is limited to ∼1.5 mm by the differential pump aperture upstream of IRU. The differential pump is needed to achieve the ultra-high-vacuum upstream of the IRU vacuum chamber required for the X-ray optical components.

The EuXFEL operates in burst mode with a burst length of up to 600 µs and a burst repetition rate of 10 Hz. Within a burst, the EuXFEL can provide X-ray pulses at MHz repetition rates, up to 4.514 MHz, derived from a main accelerator clock frequency of 1.3 GHz (Decking *et al.*, 2020 ▸). The repetition rate of X-ray pulses in a train, herein referred to as the pulse repetition rate, can be down-picked to 2.257 and 1.128 MHz, in the MHz domain and further down to the kHz domain. To record data with this pulse structure, it is necessary to trigger and synchronize the detectors with the arrival of the X-ray pulses.

In this section, we describe the instrumentation developed to enable the MHz XPCI technique at the SPB/SFX instrument. This includes an indirect X-ray microscope, sCMOS and FT-CMOS visible light detectors integrated into the EuXFEL control system, the triggering scheme with related hardware, and data acquisition by the cameras.

### 2.1. Indirect detection X-ray microscope

The key component of the MHz XPCI setup is the indirect detector, which consists of a modular X-ray microscope coupled to a fast MHz FT-CMOS camera Shimadzu Hyper Vision HPV-X2 (SHIMADZU, 2024 ▸), herein referred to as the fast camera. The available parameters for the indirect detection microscope are tabulated in Table 1 ▸. This camera records images at MHz frame rates. It is equipped with an FT-CMOS2 image sensor which has a 400 × 250 pixel format with a 32 × 32 µm pixel size. It can be operated in either full-pixel mode with a maximum recording rate of 5 × 10^6^ frames s^−1^ and a maximum of 128 frames, or half-pixel mode with a maximum recording rate of 10 × 10^6^ frames s^−1^ and a maximum of 256 frames. The spectral sensitivity of the fast camera was measured to be above 75% in the wavelength region 400–700 nm [from figure 7 of Kuroda *et al.* (2016 ▸)]. This affects the choice of the optical microscope and the scintillator in order to maximize the compatibility of the coupling between the elements. The indirect microscope relies on the conversion of X-rays into visible light using a fast decay scintillator and the subsequent projection of the light onto the fast camera. The principles and applications of indirect X-ray detection were explained in detail and further extended to micrometre scale spatial resolution by Koch *et al.* (1994 ▸, 1998 ▸). The indirect detector we have developed for MHz XPCI at EuXFEL consists of a scintillator, a 45° mirror to redirect the visible light from scintillator to the optical microscope, an exchangeable Mitutoyo objective mounted on a motorized stage and a motorized 50/50 prism beam-splitter to split the visible light into two branches to mount two independent cameras. Each branch, downstream of the beam-splitter, contains a tube lens, Mitutoyo MT-L4, which creates an image downstream at the detector planes. In standard configuration, a fast camera is mounted in one of the two branches and a slow camera in the other for alignment purposes. Alternatively, two fast cameras can be mounted on both branches. The slow camera used in the setup is an Andor Zyla 5.5 sCMOS camera (Oxford Instruments, 2023 ▸), herein referred to as the slow camera. It has 2560 × 2160 pixels (5.5 Megapixel) with a pixel size of 6.5 µm. Although the Zyla camera can run at higher repetition rates, at the EuXFEL it is triggered by a 10 Hz signal and records an integrated image of a pulse train for each trigger rising edge. The scintillators used for the microscope include cerium-doped yttrium aluminium garnet (YAG:Ce), cerium-doped yttrium aluminium perovskite (YAP:Ce), cerium-doped gadolinium aluminium gallium garnet (GAGG: Ce), cerium-doped lutetium yttrium silicate (LYSO:Ce) and CRY-60 (Crytur, 2024 ▸).

**Figure 3:**
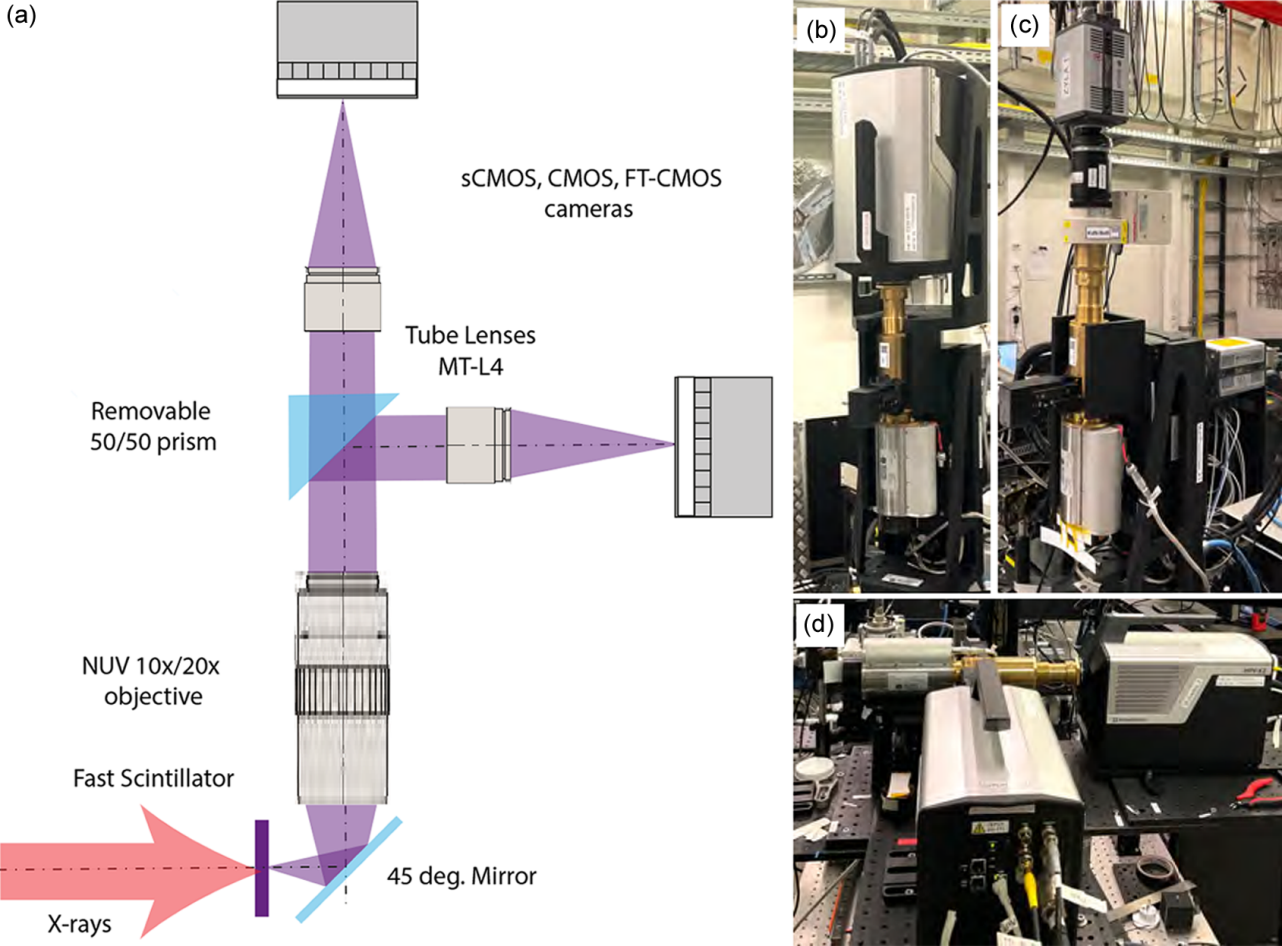
(*a*) Schematic of the modular indirect X-ray microscope. On X-ray irradiation, the luminescence from the fast scintillator is projected onto the detector using a commercial Mitutoyo NUV long-working-distance objective, 10× magnification (NA = 0.28) or 20× magnification (NA = 0.4), positioned so that the scintillator screen is in the focal plane. The objectives are motorized to find and optimize the focus easily. Downstream of the objective a removable prism beam-splitter (50/50) can be used to direct the beam to two arms of the microscope. The beam downstream of the beam-splitter is imaged onto the cameras with a tube lens, MT-L4 optimized for 266–620 nm wavelengths. The two arms of the microscope can hold (*d*) two fast cameras, or (*c*) two slow cameras, (*b*) or a combination of both. The microscope setup is equipped with *X*, *Y*, *Z* stages for precise spatial alignment of the microscope with respect to the X-ray beam. The electro-mechanical integration of the optics and cameras was carried out in close collaboration with SUNA-Precision GmbH, Germany (SUNA-Precision GmbH, 2024 ▸).

Fig. 3 ▸(*a*) shows a schematic of the indirect X-ray microscope in standard configuration using a fast and a slow camera and Fig. 3 ▸(*b*) shows the realization of this setup. In Fig. 3 ▸(*c*) the configuration with a slow camera and the Hamamatsu image intensifier for the scintillator decay time measurement is shown, and Fig. 3 ▸(*d*) shows the configuration with two fast cameras to double the camera buffer (256 frames at full resolution) or to interleave in time to double the frame rate (2.257 MHz).

The fast camera frame rate can be set with a minimum increment of 10 ns until 2 MHz. Above that, only a fixed frame rate is available with frequencies strongly mismatched to pulse repetition rates. For the pulse repetition rate of 1.128 MHz the pulse separation is 886 ns. The closest possible frame rate of the fast camera is 890 ns and the maximum exposure time of each frame at this frame rate is 590 ns. Therefore, every subsequent frame has a relative shift of 4 ns with respect to the X-ray pulse. However, the total drift of the camera frame to the X-ray pulse across the train is 512 ns, meaning there is one set fix scintillator emission within the exposure window of each frame until the last (128th) frame [Fig. 8(*b*)].

**Figure 4:**
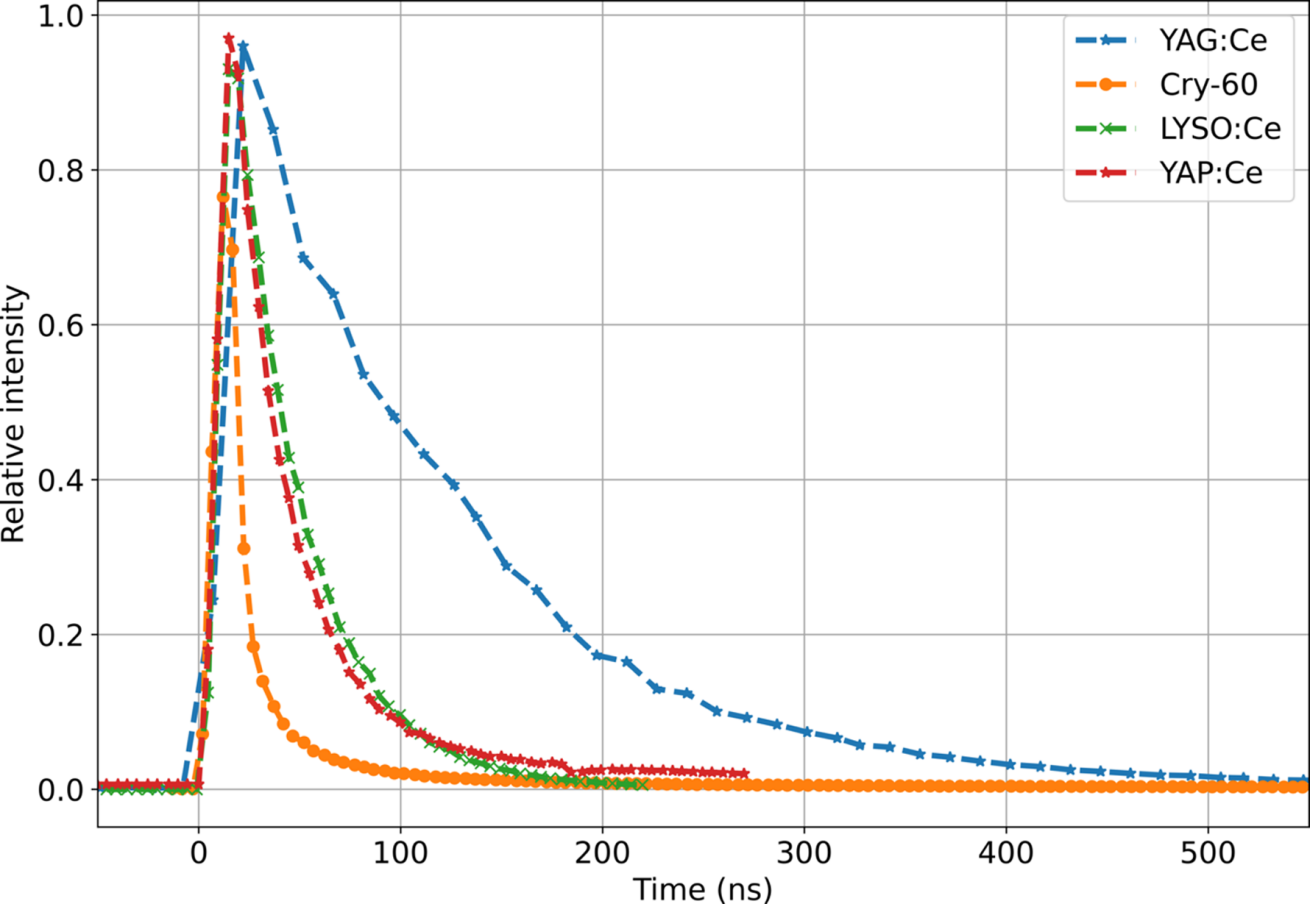
Scintillator fluorescent emission decay curves, measured using the slow camera and a gated image intensifier, show the response of the scintillator to the EuXFEL X-ray pulse.

The temporal resolution of image acquisition is determined by the pulse repetition rate and image exposure time. The exposure time is given by the X-ray pulse duration, which is extremely short, well below 100 fs. Such fast illumination ensures that each recorded frame is free of motion blur, which results in sharp images, even for phenomena as fast as shock waves. An important parameter for suitable scintillators for MHz XPCI is the fluorescent emission decay time, which has to be well below the X-ray pulse period to avoid blur or pileup. Therefore, we have examined the decay of the selected scintillators using a gated Hamamatsu image intensifier, mounted in front of the slow camera [setup shown in Fig. 3 ▸(*c*)]. We performed the measurement by shifting the gate of the image intensifier in time and recording a series of images at a given time delay with a fixed gate duration of 10 ns. After averaging the images at a given time delay, we could see that all examined scintillators have decay times well below 886 ns, proving their suitability for 1.128 MHz pulse repetition rate. The scintillators YAP:Ce, LYSO:Ce and CRY-60 show very short decay times, below 222 ns; these are plotted in Fig. 4 ▸. This makes them suitable for up to 4.514 MHz pulse repetition rate. The data presented in Fig. 4 ▸ are consistent with the findings of the previous measurements at Diamond Light Source and ESRF (Rutherford *et al.*, 2016 ▸).

### 2.2. Integration of fast and slow cameras

**Figure 5:**
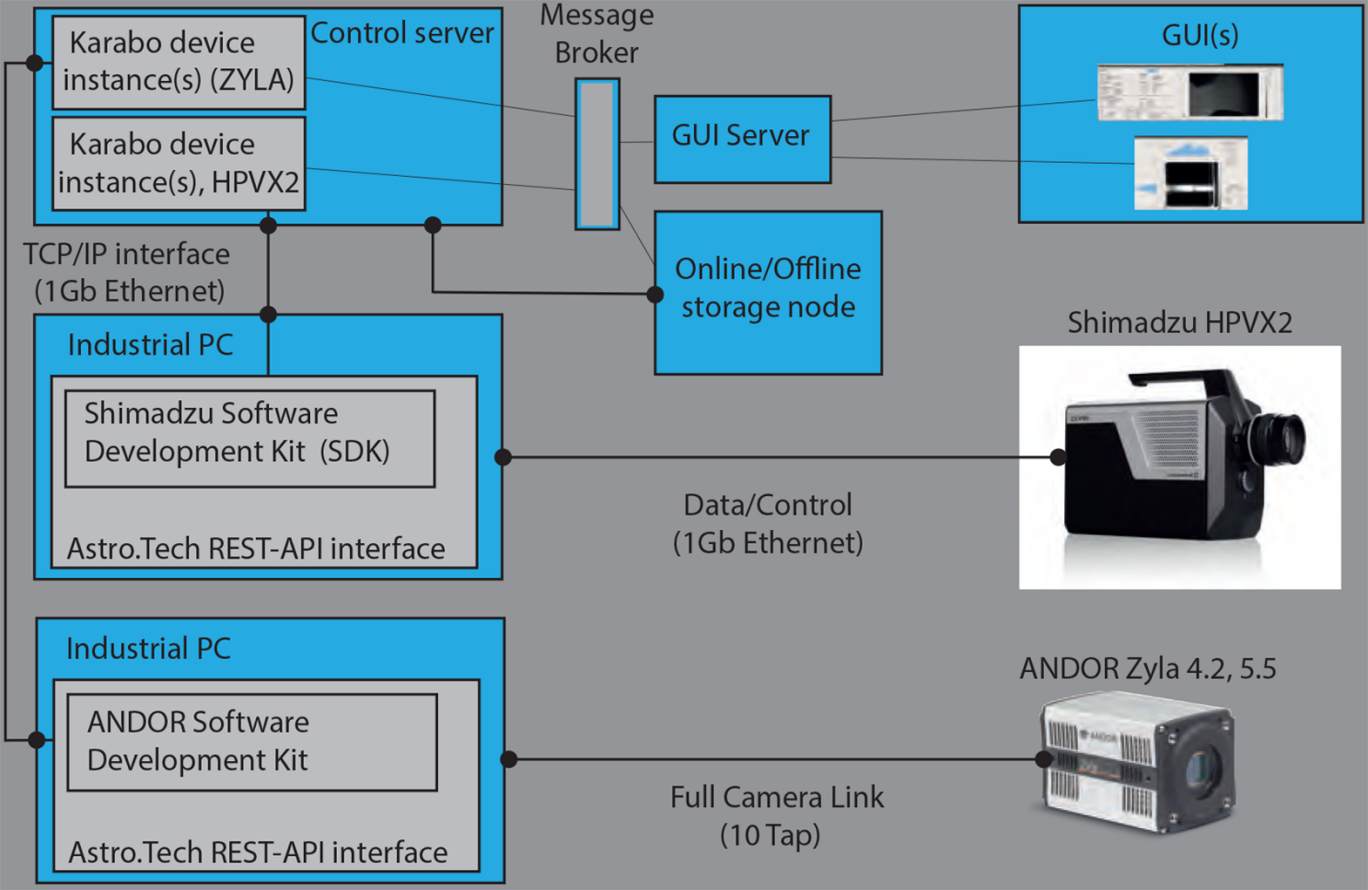
Control system view for the fast and slow cameras.

To be able to fully control the cameras and record data within the control framework of the facility, the cameras were integrated into the control system *Karabo* (Hauf *et al.*, 2019 ▸). This was achieved for both cameras by the implementation of a software layer based on a representational state transfer–application programming interface (REST-API) which communicates to the cameras via their corresponding software development kits. The REST-API exposes the camera functionality via a TCP/IP protocol and provides easy access for camera control via *Karabo*. Fig. 5 ▸ shows a schematic of how the cameras communicate with *Karabo*. The development of REST-API was outsourced to an external company, ASTRO.tech, in Germany (ASTRO.tech, 2024 ▸).

### 2.3. Synchronization and timing

For collection of the image data that are correlated to other data sources across the instrument for the same pulse train, it is crucial to synchronize the camera’s image acquisition with the arrival of X-ray pulses and align train IDs with other data sources. The EuXFEL timing and synchronization system can provide a 10 Hz signal for the pulse train and a continuous clock signal at a pulse repetition rate through microTCA boards (Branlard *et al.*, 2012 ▸; Rehlich *et al.*, 2013 ▸; Schulz *et al.*, 2019 ▸; Sato *et al.*, 2020 ▸). The slow cameras are triggered at 10 Hz (train frequency), collecting integrated images of a pulse train. Triggering and synchronization of the fast camera’s data acquisition are more complicated. The fast camera’s individual frames need to be aligned with the scintillator emissions generated at each X-ray pulse in the pulse train. Multiple fast cameras should be synchronized across the instrument to record the same pulse train, or each camera can record a different train in arbitrary offset to collect more data. The image sequence recorded by the fast camera requires at least 10 s to transfer the data through the REST-API to *Karabo* and to the data acqusition system. The next pulse train can only be recorded once the buffer is cleared.

**Figure 6:**
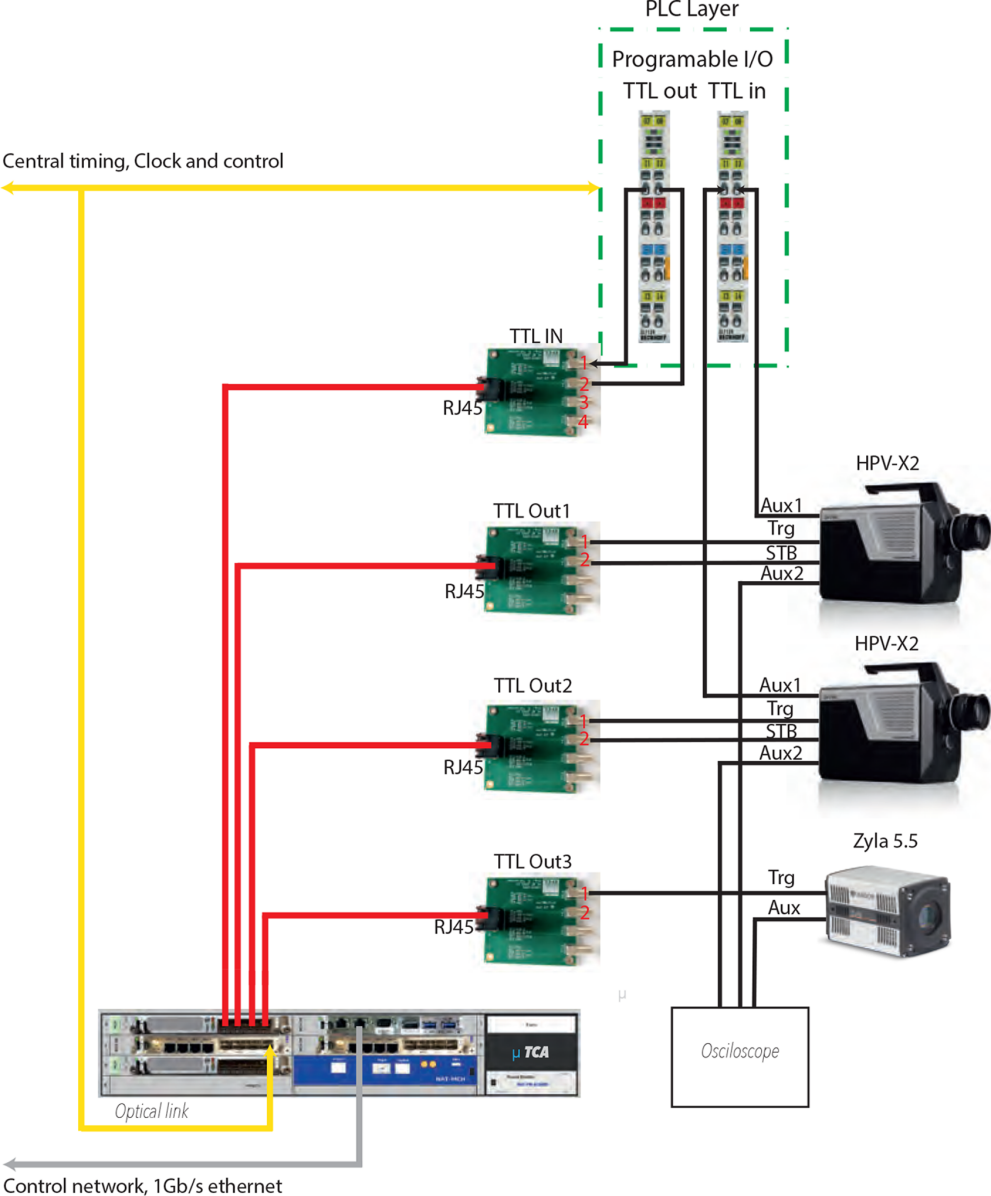
Timing signal connection diagram of fast and slow cameras. The timing and the trigger signal from the EuXFEL timing system are distributed to the microTCA unit equipped with different timing boards. Each timing board provides or receives TTL signals and offers in-built logic operations. The slow camera is triggered with a train synchronized 10 Hz trigger, which is issued before each pulse train arrival approximately 50 ms. The fast camera uses an STB synchronized signal which is generated every 15 s. The STB signal is composed of a 10 Hz trigger and a programmable TTL out signal by logical AND on the timing board. The fast camera is set to generate the output TTL signal at the start of recording of the first frame. This signal is detected by the TTL input module and only recorded data are tagged with the train ID. An oscilloscope is used to monitor these signals.

**Figure 7:**
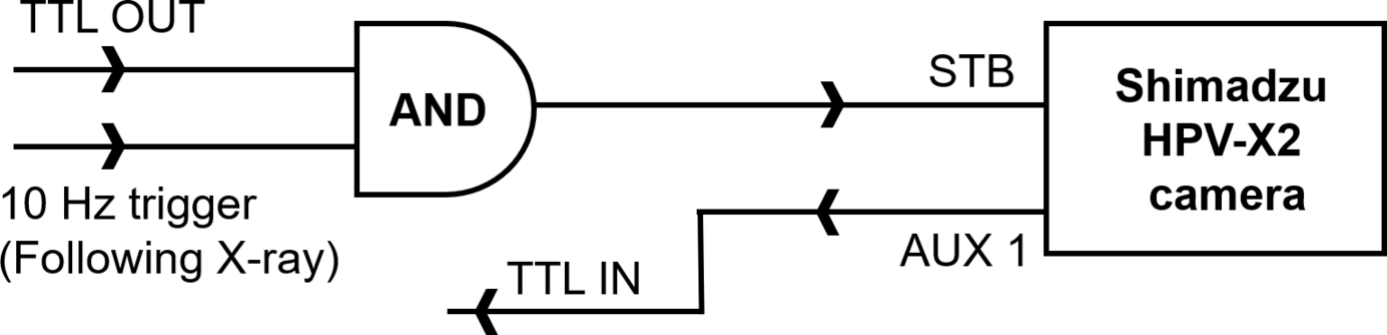
Logic diagram showing the generation of STB signal for the initiation of image acquisition by the fast camera. An enable signal from the TTL output is combined on the timing board with a 10 Hz synchronized trigger with a logical AND, and is used as the STB trigger signal for the fast camera to begin image acquisition. Once the fast camera begins the image acquisition, it generates a TTL signal from on AUX1 TTL output. This signal is connected to the TTL input of the PLC device where the data are tagged with a train ID upon detection of a rising edge.

**Figure 8:**
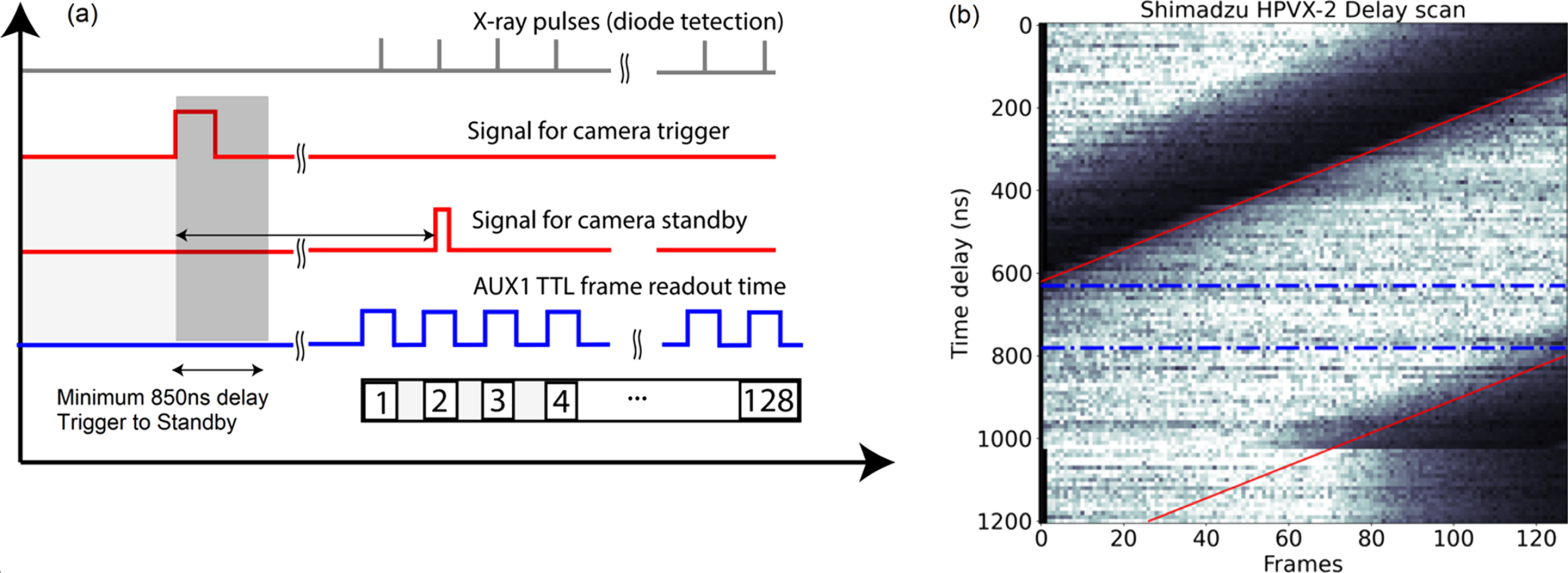
(*a*) Timing signal configuration for image acquisition for one Shimadzu HPV-X2 camera and (*b*) plot of STB trigger delay versus mean intensity of each frame across the buffer. The slope indicated by the red lines in the graph represents the camera clock mismatch with the EuXFEL clock. The pulse separation at 1.128 MHz is 886 ns. However, the minimum increment for shifting acquisition available on the camera is 10 ns, resulting in a closest frame rate of 890 ns. This causes a drift of each subsequent acquisition window with respect to the scintillator emission by 4 ns and thus the total drift across the camera buffer is 512 ns. With a maximum possible acquisition time for each frame of 590 ns, the buffer can be aligned to record signal on all 128 frames. The delay range highlighted between the two dotted blue lines shows the optimal delay position where full buffer stays synchronized to all 128 X-ray pulses.

A synchronization scheme based on EuXFEL’s timing and synchronization system was developed to synchronize the fast cameras with X-rays and to each other. Fig. 6 ▸ shows the timing and synchronization system for fast cameras. The signal generated by the programmable logic controller (PLC) input/output (I/O) modules is connected to the input timing card of the microTCA board, acting as a programmable enable signal. This synchronized signal is combined with a 10 Hz signal using a logical AND in the microTCA board. The resulting signal is then connected to the camera standby (STB) trigger to initiate data acquisition every 15 s. A logical diagram of the timing signal for triggering the fast camera is shown in Fig. 7 ▸. All these timing signals can also be monitored via an oscilloscope. Fig. 8 ▸ shows the configuration of timing signals for two fast cameras and one slow camera along with an STB delay scan performed to align the acquisition windows to the scintillator emissions. The same triggering sequence is applied for other fast cameras.

Each X-ray pulse train is tagged with a train ID. This tagging is particularly important for data acquired by the fast cameras in order to facilitate the correlation with datasets from other sources. To tag the data from the fast camera with train IDs, we explored two methods. The first is based on the system clock of an industrial PC, synchronized with a network time protocol server located in the control network. This approach did not provide accurate timing because of high latency of time synchronization of the operating system (Windows 7), resulting in fluctuations of the train ID in the range ±7. The second approach is based on the generation of a rising-edge output TTL signal from the FT-CMOS camera (AUX 1) generated at the time of recording the first frame. This signal is connected to a 5 V TTL digital input of a PLC module, and the train ID is attached when a rising edge is detected. We align the rising edge of the output TTL signal from the fast camera (AUX 2, copy of AUX 1) to the camera buffer signal by monitoring it with an oscilloscope. This second approach gave reliable results with no fluctuations of train IDs.

### 2.4. MHz pulse resolved beam diagnostics using the fast camera

**Figure 9:**
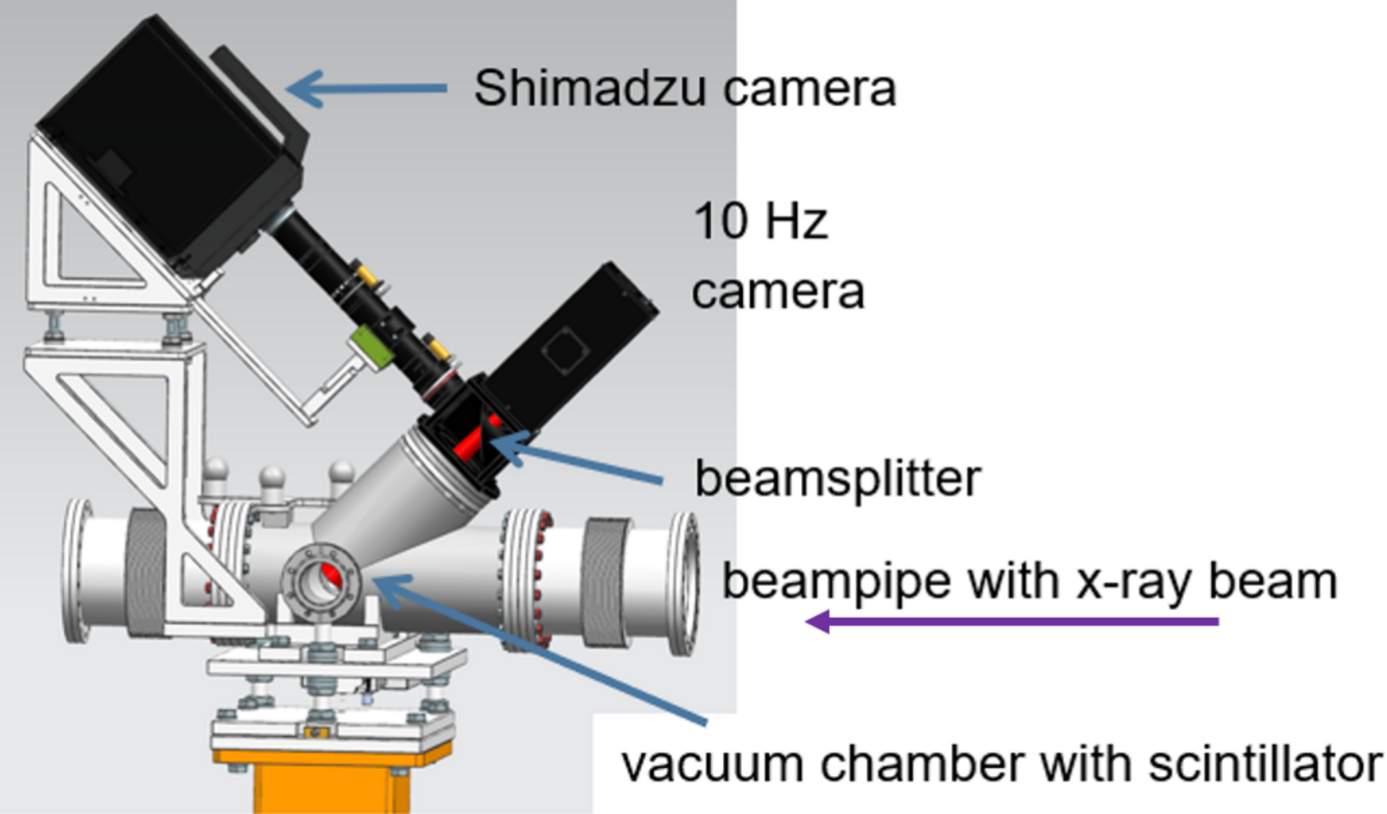
Shimadzu HPV-X2 camera used for beam monitoring: the fast camera allows intra-train recording of the beam at MHz rates. A slow camera simultaneously views the beam via an optical beam-splitter.

The fast camera, integrated into the timing and control system, was also used to enable the photon diagnostics at MHz rates. A pulse resolved photon beam monitoring setup was installed in the SASE1 tunnel upstream of the SPB/SFX instrument. This monitoring system consists of the fast camera, a YAG:Ce scintillator, a 50/50 beam-splitter and a Navitar lens (Resolv4K). Fig. 9 ▸ shows the implemented setup. The field of view depends on the zoom settings of the lens; it ranges from 50 × 31 to 7.2 × 4.5 mm with pixel sizes of 125 and 18 µm, respectively, in the scintillator plane. The beam-splitter allows simultaneous recording by a standard slow camera with a wider field of view and higher spatial resolution (Koch *et al.*, 2019 ▸). This setup facilitates the spatial characterization of the photon beam at MHz rates. The fast camera installed for photon beam diagnostics can also be synchronized with the MHz XPCI setup in the SPB/SFX experimental hutch using the synchronization scheme mentioned in Section 2.3. This enables two-plane imaging of the photon beam at MHz repetition rates. This MHz beam diagnostic along with the MHz X-ray microscope were used in multiple beam studies at EuXFEL to better characterize the pulse-to-pulse beam properties (Guest *et al.*, 2022 ▸, 2023 ▸).

## 3. Applications of MHz XPCI

The developments detailed above allow MHz XPCI of stochastic phenomena with photon energies up to 24 keV. This section describes some applications where MHz XPCI was used to study stochastic phenomena at the SPB/SFX instrument.

### 3.1. Foaming process in aluminium

**Figure 10:**
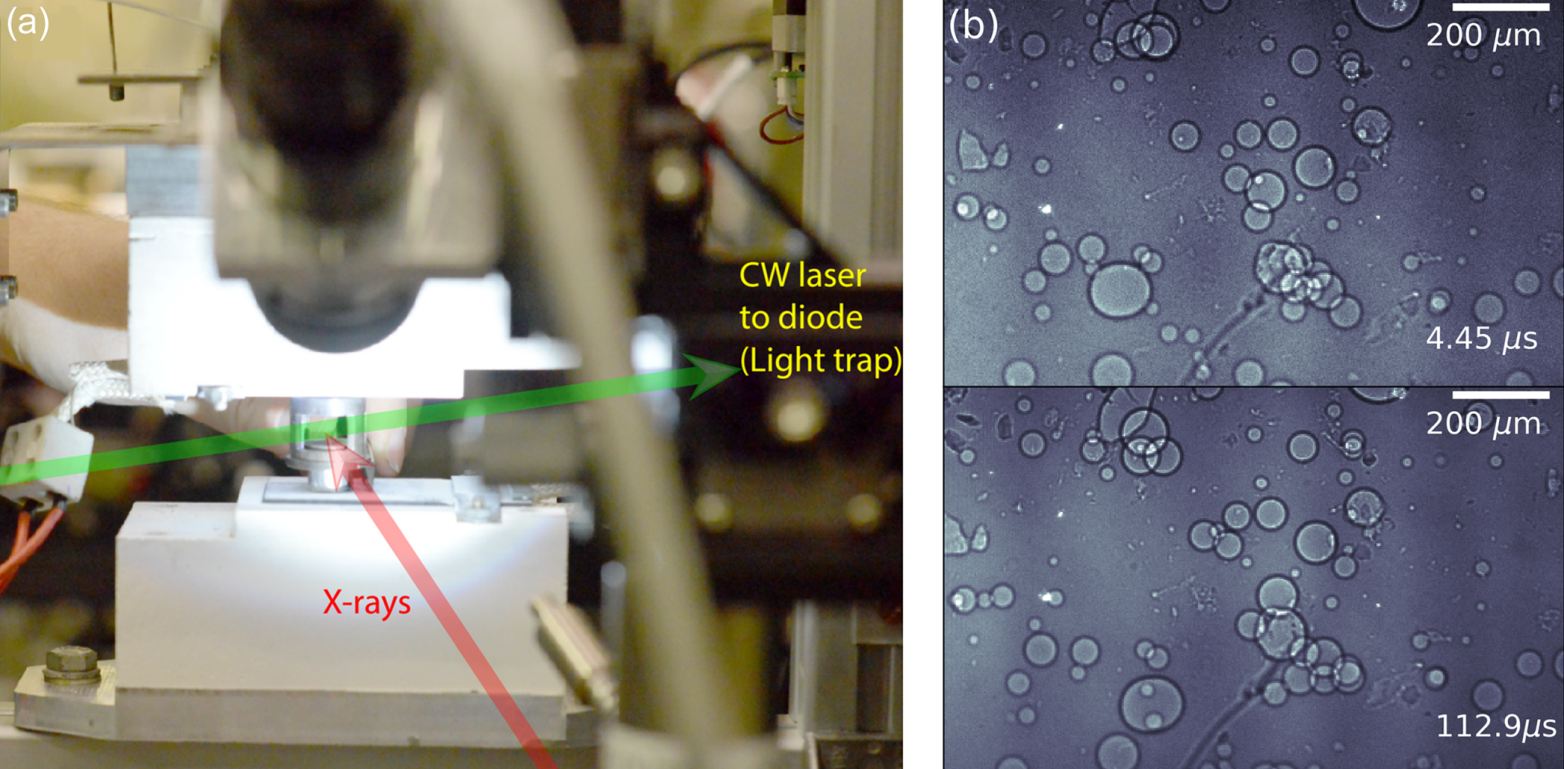
(*a*) Setup for the aluminium foaming process and (*b*) images of Al foam dynamics at different times within one pulse train.

The dynamics of liquid aluminium based foam have been studied using MHz XPCI to capture bubble coalescence. Bubble coalescence is a fast process that occurs on a timescale on the order of nanoseconds to microseconds, depending on the bubble size (García-Moreno *et al.*, 2019 ▸; Zhang *et al.*, 2021 ▸). Images acquired were recorded at a photon energy of 24 keV, a pulse energy of nearly 800 µJ and a repetition rate of 1.128 MHz. This photon energy was reached by the accelerator running at an energy of 17.3 GeV. The measurement used the MHz X-ray microscope discussed in Section 2.1 with a 10× objective, giving an effective pixel size of 3.2 µm and a 20 µm-thick Ce:YAG scintillator. Fig. 10(*a*) ▸ shows the setup for the aluminium foaming experiment. By heating the sample to 600°C, the blowing agent (TiH_2_) releases hydrogen and increases the pressure in the crucible, which leads to the seal breaking. Immediately afterwards, the growing liquid metal foam escapes through the opening and triggers the light trap. This then simultaneously triggers the X-ray shutter to allow X-rays to pass through the sample and the fast camera to image the evolving foaming process. Some images of the evolving foam in the early stage are shown in Fig. 10(*b*) ▸.

### 3.2. Dynamics of exploding droplets

**Figure 11:**
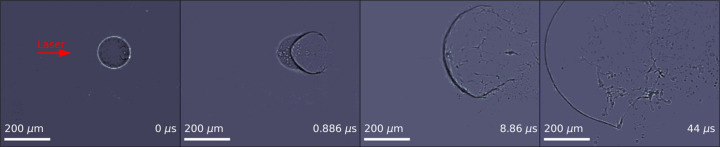
One realization of different stages of a laser-induced explosion of a water droplet recorded using MHz XPCI. The images were denoised using a robust principal component analysis (Candès *et al.*, 2011 ▸; Brunton & Kutz, 2022 ▸).

The dynamics of laser-induced droplet fragmentation were studied using MHz XPCI (Reuter *et al.*, 2023 ▸). A clear view of the droplet and the cavitation developing over time in its interior was obtained. Such a system is not transparent to visible light due to the opacity caused by highly curved interfaces. The water droplets were injected and trapped in nodes of standing wave pattern of an acoustic levitator, and the focused beam of a pump–probe laser [1030 nm, 850 fs, 1 mJ (Koliyadu *et al.*, 2022 ▸)] with the focal spot placed at the droplet position was used to initiate the droplet explosion. The temporal evolution of the droplet explosion was imaged using 12 keV X-rays and recorded using two MHz X-ray microscopes at a 1.128 MHz pulse rate, aligned sequentially in time to capture dynamics on longer timescales. Both microscopes were equipped with a 10× Mitutoyo long-working-distance objective enhanced for the near-ultraviolet range with a 0.28 NA and a 20 µm-thick Ce:YAG scintillator. This produces an effective pixel size of 3.2 µm. Fig. 11 ▸ shows several stages in the evolution of an exploding droplet across a pulse train.

### 3.3. Vortex cavitation in a Venturi tube

**Figure 12:**
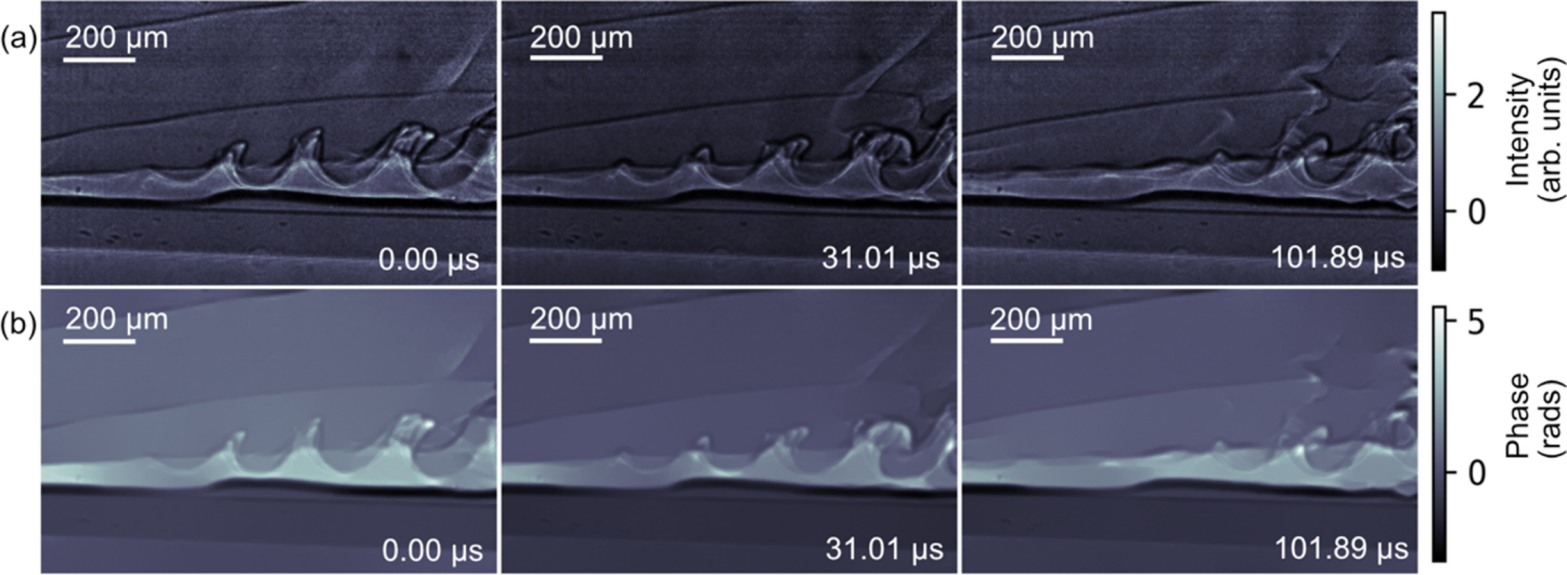
Stages of Kelvin–Helmholtz instability formation in a Venturi tube imaged at 1.128 MHz: (*a*) the flat-field-corrected images at three different time points and (*b*) the images after phase retrieval methods based on the alternating direction method of multipliers (Villanueva-Perez *et al.*, 2017 ▸). Videos S1 and S2 of the supporting information show the flat-field-corrected and phase-retrieved data, respectively.

The MHz XPCI setup was used to study vortex cavitation phenomena in a Venturi tube, known for its aggressive behaviour near surfaces (Soyama *et al.*, 2023 ▸). An experimental setup with a Venturi tube was constructed and used to generate a water flow through the Venturi, and the turbulent flow across the tube was imaged. The 9.3 keV X-rays transmitted through the Venturi tube were captured by an MHz X-ray microscope at 1.128 MHz, equipped with a 10× Mitutoyo long-working-distance objective enhanced for the near-ultraviolet with a 0.28 NA and a 20 µm-thick Ce:YAG scintillator. Fig. 12 ▸ shows a few stages in the evolution of the cavitation formation in the region where Kelvin–Helmholtz instabilities (Podbevšek *et al.*, 2021 ▸; Boček *et al.*, 2024 ▸) are formed with observed velocities of 100 m s^−1^.

## 4. Conclusions

We have described and summarized the instrumentation developed for the MHz XPCI method at the SPB/SFX instrument of EuXFEL. This development also enabled the realization of MHz X-ray beam characterization diagnostics at EuXFEL. The various components of the microscope, the integration of cameras into the control system, and the timing and synchronization system have been discussed in detail. Furthermore, we showed examples of applications such as the foaming process in aluminium, the dynamics of exploding droplets and vortex cavitation in a Venturi tube. The implementation of MHz XPCI at EuXFEL opens up new possibilities for academic and industrial research for broad applications. This method is now available to a user community through the standard proposal process of EuXFEL.

## Supplementary Material

Flat-field-normalized and phase-retrieved video sequence of Kelvin-Helmholtz instability. DOI: 10.1107/S160057752400986X/yi5159sup1.mp4

Flat-field normalized video sequence of Kelvin-Helmholtz instability. DOI: 10.1107/S160057752400986X/yi5159sup2.mp4

## Figures and Tables

**Table 1:** Available parameters for the indirect detection microscope at EuXFEL

	Fast camera	Slow camera
Camera sensor	FT-CMOS2 image sensor	sCMOS image sensor
Pixel size (µm)	32 × 32	6.5 × 6.5
Sensor size (pixels)	400 × 250	2560 × 2160 pixels
Field of view with 10× objective (mm)	1.28 × 0.8	1.6 × 1.4
Pixel size with 10× magnification (µm)	3.2 × 3.2	0.65 × 0.65
Frame rate (MHz)	Up to 1.1	Up to 10
Maximum frames per pulse train (frames)	128	1
